# Comprehensive analysis of potential immunotherapy genomic biomarkers in 1000 Chinese patients with cancer

**DOI:** 10.1002/cam4.2381

**Published:** 2019-07-04

**Authors:** Yuan‐sheng Zang, Chun Dai, Xiaoman Xu, Xin Cai, Guan Wang, Jinwang Wei, Angela Wu, Wending Sun, Shunchang Jiao, Qiang Xu

**Affiliations:** ^1^ Department of Medical Oncology Changzheng Hospital, Naval Medical University Shanghai China; ^2^ GenomiCare Biotechnology Co. Ltd. Shanghai China; ^3^ Department of Medical Oncology Chinese PLA General Hospital Beijing China

**Keywords:** Chinese patients with cancer, dMMR, MSI, PD‐L1 amplification, TMB, WES

## Abstract

**Background:**

Tumor mutation burden (TMB), DNA mismatch repair deficiency (dMMR), microsatellite instability (MSI), and PD‐L1 amplification (PD‐L1 AMP) may predict the efficacy of the PD‐1/PD‐L1 blockade. With the broadening landscape of immunotherapy use, it is important to identify patients who are likely to benefit from the therapy. This study aimed to characterize the distributions of these biomarkers and explore the relationships among these biomarkers for Chinese patients with cancer.

**Methods:**

In this study, we examined the aforementioned biomarkers in more than 1000 Chinese patients with cancer. These biomarkers were determined based on whole‐exome sequencing (WES) of tumor/blood samples.

**Results:**

Of the 953 samples from Chinese cancer patients assessed in this study, 35% exhibited high TMB (TMB‐H), 4% were positive for high MSI (MSI‐H), dMMR occurred in 0.53%, and PD‐L1 AMP was positive in 3.79%. We found higher rates of TMB‐H among hepatocellular carcinoma, breast cancer, and esophageal cancer patients than was reported for The Cancer Genome Atlas (TCGA) data. Lung cancer patients with *EGFR* mutations had significantly lower TMB values than those with wild‐type *EGFR*, and increased TMB was significantly associated with dMMR in colorectal cancer (CRC). The frequency of tumors with MSI‐H was the highest in CRC and gastric cancer. PD‐L1 AMP occurred most frequently in lung squamous cell carcinoma and HER2‐positive breast cancer. While MSI and dMMR are associated with higher mutational loads, correlations between TMB‐H and other biomarkers, between MSI‐H and dMMR, and between PD‐L1 AMP and other biomarkers were low, indicating different underlying causes of the four biomarkers.

**Conclusion:**

The results reveal the frequency of these biomarkers in different malignancies, with potential implications for PD‐1/PD‐L1 blockade use for Chinese patients with cancer.

## 1 INTRODUCTION

The PD‐1/PD‐L1 blockade has become a powerful approach for treating multiple types of cancer. Many patients benefit from such treatments, exhibiting not only a higher objective response rate, but also durable remission for many years.^1^, ^2^, ^3^, ^4^, ^5^, ^6^ While treatment with PD‐1/PD‐L1 blockade can be highly effective, not every patient or cancer type responds to these inhibitors, and some patients experience hyperprogression after immunotherapy.^7^ Identifying which patients may benefit from these inhibitors is one of the most significant current challenges. Therefore, the identification of precise biomarkers to predict the efficacy of PD‐1/PD‐L1 blockade is critical.

Several studies have analyzed, in detail, the correlation between tumor mutation burden (TMB) and the efficacy of immunotherapy, showing a high association between TMB and treatment efficacy.^8^, ^9^, ^10^ A study involving 151 cancer patients further confirmed a linear relationship between TMB and the clinical outcome of immunotherapy.^9^ Analysis of almost 1700 cancer patients receiving at least one dose of PD‐1/ PD‐L1 blockade showed that 20 percent of the patients with the highest TMB in each cancer type had a better overall survival.^11^ Furthermore, the US Food and Drug Administration (FDA) approved this treatment for unresectable or metastatic solid tumors in patients with high MSI (MSI‐H) or dMMR based on five clinical trials. These trials across 15 cancer types involving 149 patients with high microsatellite instability (MSI‐H) or DNA mismatch repair deficiency (dMMR) reported complete or partial response to pembrolizumab in 39.6% of patients. Moreover, in 78% of the responding patients, the response lasted for 6 months or longer.^12^, ^13^

PD‐L1 (CD274) amplification (PD‐L1 AMP) was also identified as a predictor of response to PD‐1/PD‐L1 blockade therapy. In classical Hodgkin's lymphoma, 97% of patients exhibit PD‐L1 AMP.^14^ Compared with other cancers, patients with Hodgkin's lymphoma exhibit a higher overall response rate, reaching 69%.^15^, ^16^ In rare metastatic basal cell carcinoma, PD‐L1 AMP is also related to response to nivolumab.^17^ Thus, there is a strong correlation between PD‐L1 AMP and response to the PD‐1/PD‐L1 blockade.

Given that the above markers are potential biomarkers of PD‐1/PD‐L1 blockade efficacy, we aimed to characterize the distributions of these biomarkers in more than 1000 Chinese patients with cancer using exome profiling data. We also explored the relationships among TMB, MSI‐H, dMMR, and PD‐L1 AMP.

## 2 METHODS

### 2.1 Patient characteristics

We collected over 1000 Chinese cancer specimens from more than 70 hospitals in 20 Chinese provinces from October 2015 to March 2016. In total, there were 1179 samples, including 524 biopsy samples and 655 formalin‐fixed, paraffin‐embedded samples. Blood samples were also collected as controls. All procedures followed the Molecular Pathology Clinical Practice Guidelines and Reports^18^ and were performed in accordance with the 1964 Declaration of Helsinki.

### 2.2 WES analysis

The complete exomes of tumor samples and matched blood samples were sequenced for each patient. DNA was fragmented and hybridized to the SureSelect Human All Exome Kit V5 (Agilent Technologies, Santa Clara, CA, USA), containing exon sequences from 27 000 genes. Exome shotgun libraries were sequenced on the Illumina Xten platform, generating paired‐end reads of 150 bp at each end. Image analysis and base calling were performed with CAVSAVR (Illumina, San Diego, CA, USA) using default parameters. Sequencing adaptors and low‐quality reads were removed to obtain high‐quality reads. These were aligned to the NCBI human reference genome hg19 using the Burrows‐Wheeler Aligner alignment algorithm.

We used the Genome Analysis Toolkit (GATK, version 3.5) to process reads. Localized insertion‐deletion (indel) realignments were performed using GATK. GATK Realigner Target Creator was used to identify regions for realignment. For single‐nucleotide variant (SNV) calling, the MuTect algorithm was applied to identify candidate somatic SNVs in tumors by comparison with the matched control blood sample from each patient. SNV annotation was performed using ANNOVAR. We used dbNSFP31 to predict nonsynonymous mutations in the encoded proteins. For dMMR, we identified SNVs in *MLH1*, *MSH2*, *MSH6,* and *PMS2*.

For indel detection, tumor and blood samples were analyzed with VarscanIndel. Candidate somatic indels were identified based on the following criteria: (a) supported by at least five reads and (b) the number of supporting reads divided by the maximum read depth at the left, and right breakpoint positions were >0.05. All somatic indel calls were manually checked with the Integrative Genomics Viewer.

The CNVnator software tool was used to detect somatic copy number variations.^19^ All parameters were set to their defaults for filtering samples, and the bin size was set to 50‐60 according to the average coverage depth. A PD‐L1 copy number ≥3 was defined as PD‐L1 AMP.

### 2.3 TMB evaluation

Tumor mutation burden was defined by the total number of somatic nonsynonymous mutations (NSM), which was determined by comparing sequence data between tumor tissues and matched blood samples using a previously described method.^20^ We defined the higher tertile of the TMB of each cancer type as the threshold for TMB‐H according to the method of a prior study.^21^

### 2.4 MSI evaluation

All autosomal microsatellite tracts containing five or more repeating subunits 1‐5 bp in length in GRCh37/hg19 were identified using MISA (http://pgrc.ipk-gatersleben.de/misa/misa.html). Detailed calculations were performed as described previously.^22^ Patients were classified into the microsatellite stable (MSS), low MSI (MSI‐L), and MSI‐H groups with 0%‐1%, 1%‐3.5%, and ≥3.5% unstable microsatellite sites, respectively, according to a previous method.^23^

### 2.5 Statistical analysis

Summary statistics were used with the mean or median for continuous variables. The Mann‐Whitney *U* test was used to assess non‐normally distributed variables TMB *P*‐values < 0.05 were considered significant. Statistical analyses were carried out using GraphPad Prism version 7.0 (San Diego, CA).

## 3 RESULTS

### 3.1 Cohort description

**Figure 1:**
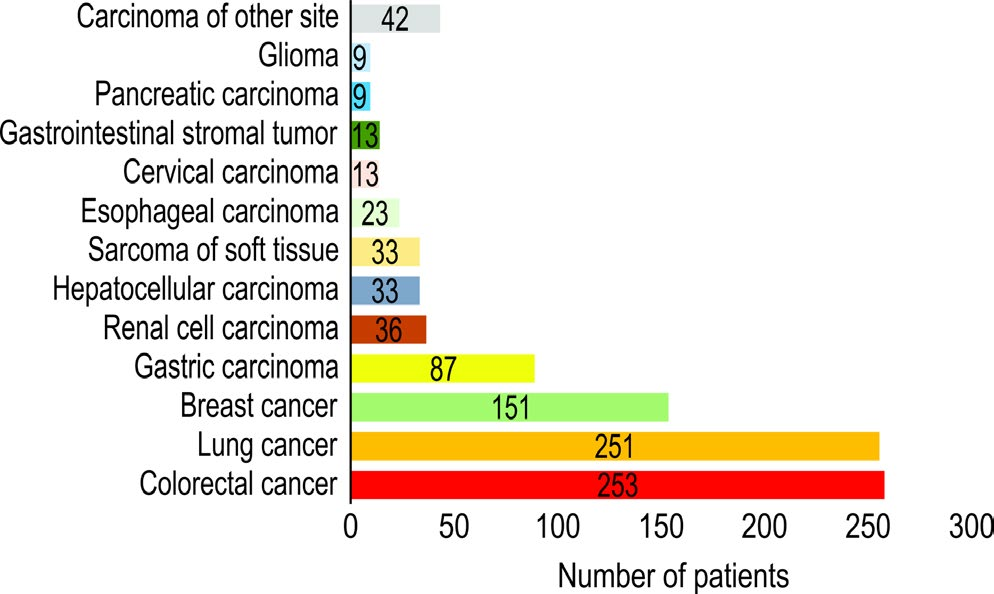
Distribution of cancer types among 953 samples from Chinese patients analyzed using whole‐exome sequencing

We collected 1197 tumor samples from Chinese patients. Due to unqualified samples and sequencing failures, 953 samples were successfully sequenced using whole‐exome sequencing (WES). These tumors encompassed four principal tumor types, including colorectal cancer (CRC) and lung, breast, and gastric cancer, accounting for 78% of the tumors. There were also more than eight other tumor types (Figure 1). Based on the cancer distribution in China,^24^ we collected more lung adenocarcinoma (LUAD) cases (n = 172) than lung squamous cell carcinoma (LUSC) cases (n = 42) and other subtypes (n = 37). Among breast cancer cases, the number of patients with HER2^+^ (n = 34), HER2^−^ (n = 49), and HER2 status unknown (n = 68) cancer were similar.

### 3.2 TMB profiling

**Figure 2:**
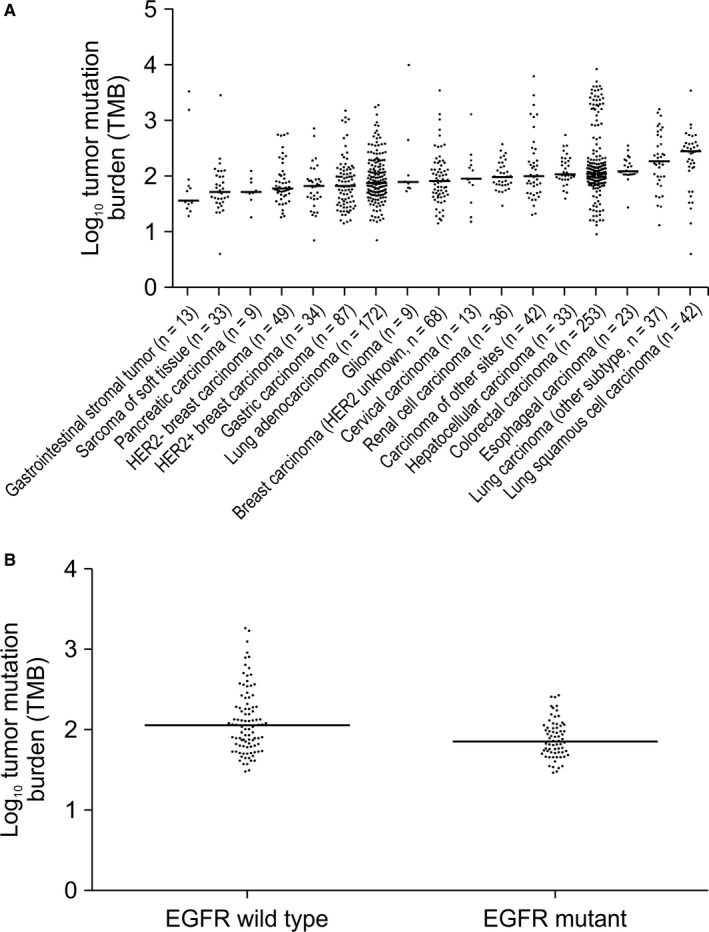
Characterization of tumor mutation burden (TMB). A, The median number of somatic nonsynonymous mutations (log_10_‐transformed) was plotted for each cancer type after analysis of 953 samples from Chinese patients. The difference in median TMB between lung adenocarcinoma (LUAD) and lung squamous cell carcinoma (LUSC) was statistically significant (*P* < 0.0001 by Mann‐Whitney *U* test). B, The median number of somatic nonsynonymous mutations (log_10_‐tranformed) was plotted for LUAD patients with or without *EGFR* mutation (*P* = 0.0039 by Mann‐Whitney *U* test)

We analyzed the TMB of the 953 Chinese patients with cancer. The median TMB of each cancer type ranged from 36 to 273 NSM, with an overall median of 95 NSM, and 54 patients exhibited NSM values > 1000. The two cancer types with the highest TMB were lung cancer and CRC, with median TMB of 176 and 108 NSM, respectively. Hepatocellular carcinoma, renal cell carcinoma, and glioma also had high TMB values, while the lowest TMB was observed among gastrointestinal stromal tumors. We also found that different tumor subtypes exhibited different TMB, especially in lung cancer: LUSC cases had median TMB values more than three times higher than those in LUAD cases (273 vs 74 NSM, respectively) (Figure 2A).

Although PD‐1/PD‐L1 blockade has been approved in Non‐small cell lung cancer (NSCLC), some clinical trials have reported that NSCLC patients with *EGFR* mutations do not benefit from this therapy and that it may even lead to a more rapid disease progression.^1^, ^7^, ^25^ Therefore, we compared the median TMB of LUAD patients with mutant (n = 77) and wild‐type (n = 99) *EGFR* genes. Patients with *EGFR* mutations had significantly lower median TMB compared with those with wild‐type *EGFR* (74 vs 113 NSM, respectively; *P* = 0.0039) (Figure 2B).

### 3.3 MSI and dMMR distributions

**Figure 3:**
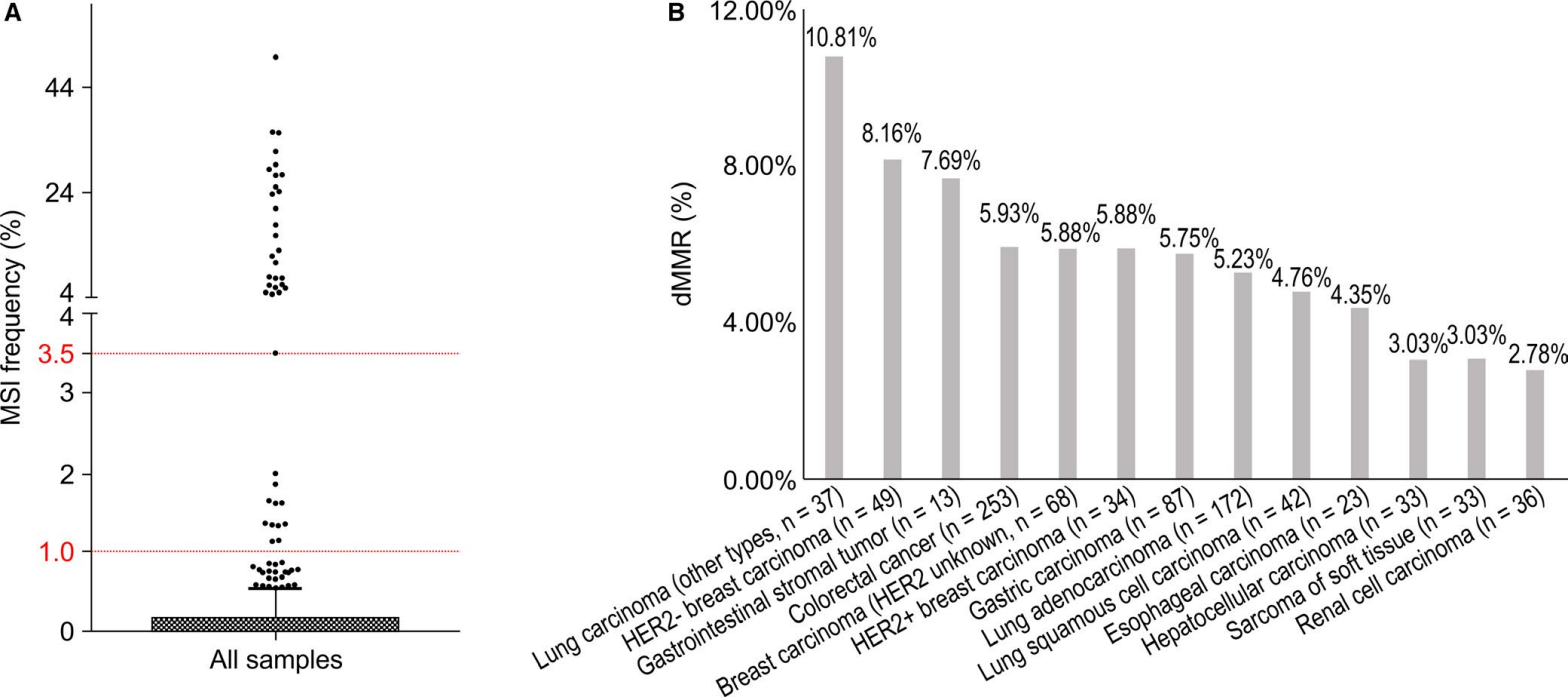
MSI and dMMR distribution in 953 Chinese patients. A, MSI distribution. Patients in the MSS, MSI‐L, and MSI‐H groups exhibited 0%‐1%, 1%‐3.5%, and ≥3.5% unstable microsatellite sites, respectively. B, dMMR distribution. dMMR, DNA mismatch repair deficiency; MSI, microsatellite instability; MSI‐H, high MSI; MSI‐L, low MSI; MSS, microsatellite stable

We investigated the microsatellite instability (MSI) and dMMR distributions in Chinese patients with cancer. Tumors were classified into three groups based on the proportion of unstable microsatellites, consistent with previous reports^23^: MSS, below 0%‐1% (n = 597); MSI‐L, 1%‐3.5% (n = 12); and MSI‐H, ≥3.5% (n = 28) (Figure 3A). The frequency of tumors classified as MSI‐H was 8.3% for CRC and 2.3% for gastric cancer, while lung and breast cancer exhibited MSI‐H frequencies below 3%. Gastrointestinal stromal tumors exhibited a high frequency of dMMR, while those of hepatocellular carcinoma, sarcoma of soft tissue, and renal cell carcinoma were among the lowest. The dMMR frequency of lung carcinoma (other types) was nearly double those in LUAD and LUSC. HER2^−^ breast carcinoma exhibited a higher dMMR frequency than HER2^+^/HER2 unknown breast carcinoma (Figure 3B).

### 3.4 PD‐L1 AMP distribution

**Figure 4:**
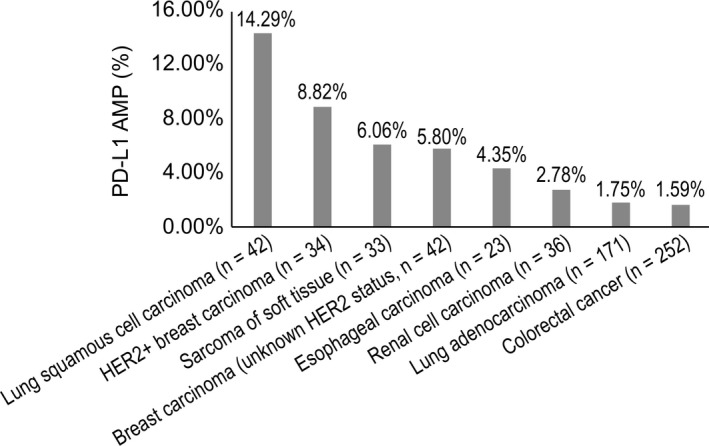
PD‐L1 amplification frequency across eight major tumor types

We analyzed the distribution of PD‐L1 AMP among Chinese patients with cancer. We observed that PD‐L1 AMP occurred most frequently in LUSC (14.3%), and HER2^+^ breast cancer (8.8%), and breast cancer with unknown HER2 status (5.8%). In contrast, LUAD and CRC had lower rates of PD‐L1 AMP at 1.75% and 1.59%, respectively (Figure 4). Thus, LUAD and LUSC exhibit large differences in levels of PD‐L1 AMP. Moreover, we compared the frequencies of the biomarkers for immunotherapy in the Chinese population with those of Western populations and TCGA data (Table 1).

**Table 1:** Comparison of the frequencies of biomarkers for immunotherapy in the Chinese population with those of Western populations and TCGA data

TMB (NSM)		Reference^26^, ^27^	
Pancreatic carcinoma	51	Pancreatic adenocarcinoma	45
Breast carcinoma	66	Breast carcinoma	33
Gastric carcinoma	66	Stomach adenocarcinoma	179
Glioma	77	Glioma	31
Cervical carcinoma	88	Cervical squamous cell carcinoma	46
Renal cell carcinoma	94	Renal cell carcinoma	38
Hepatocellular carcinoma	106	Hepatocellular carcinoma	39
Colorectal Carcinoma	108	Colon cancer	315
Esophageal carcinoma	120	Esophageal carcinoma	75
Lung squamous cell carcinoma	273	Lung squamous cell carcinoma	175
Lung adenocarcinoma	74	Lung adenocarcinoma	155

MSI‐H (%)		Reference^28^	
Colorectal Carcinoma	8.30	Colorectal Carcinoma	13
Gastric carcinoma	2.30	Gastric carcinoma	22
Lung cancer	0.40	Lung cancer	2
Breast carcinoma	0.66	Breast carcinoma	1

dMMR (%)		Reference^12^	
Gastric carcinoma	5.75	Gastric adenocarcinoma carcinoma	≈9
Breast carcinoma	6.62	Breast carcinoma	1
Hepatocellular carcinoma	3.03	Hepatocellular carcinoma	3
Colorectal Carcinoma	5.93	Colorectal adenocarcinoma	6
Esophageal carcinoma	4.35	Esophageal carcinoma	1
Non‐small cell lung cancer	5.14	Non‐small cell lung cancer	1

PD‐L1 amplification (%)		Reference^29^	
Lung squamous cell carcinoma	14.29	Lung squamous cell carcinoma	>15
Sarcoma of soft tissue	6.06	Sarcoma	>15
Renal cell carcinoma	2.78	Renal clear carcinoma	<5
Lung adenocarcinoma	1.75	Lung adenocarcinoma	10‐15
Colorectal cancer	1.59	Colorectal adenocarcinoma	>15
Breast carcinoma	8.54	Breast invasive carcinoma	>15

Abbreviations: dMMR, DNA mismatch repair deficiency; MSI‐H, high MSI; NSM, nonsynonymous somatic mutations; TMB, tumor mutation burden.

### 3.5 Relationships among biomarkers

**Figure 5:**
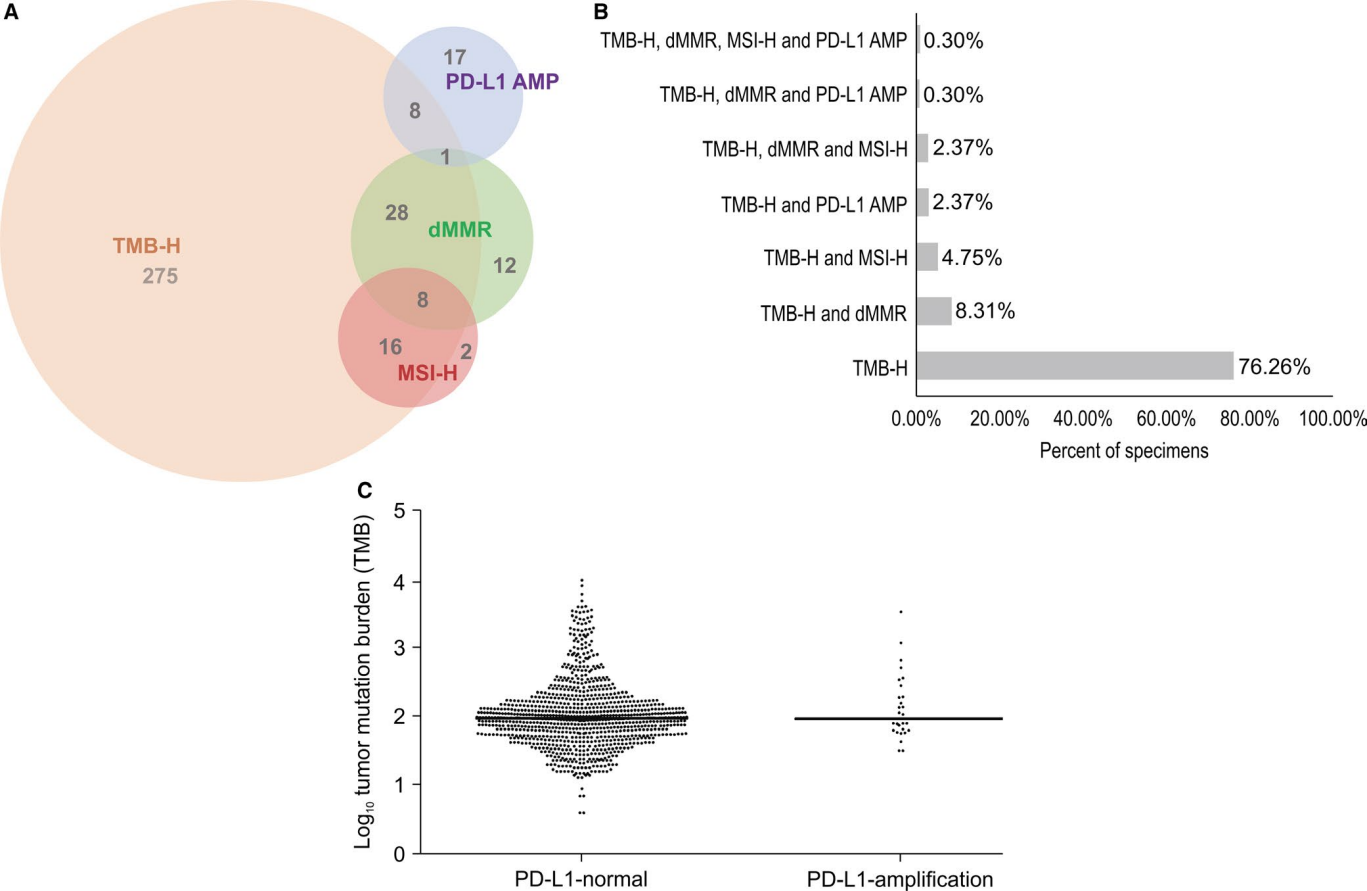
Relationship between TMB‐H and MSI, dMMR, and PD‐L1 AMP across cancer types in Chinese patients. A, Overlapping distributions of TMB, MSI, dMMR and PD‐L1 AMP. B, Proportions of TMB‐H patients positive for other biomarkers. C, TMB in all samples with (n = 28) or without (n = 922) PD‐L1 AMP. dMMR, DNA mismatch repair deficiency; MSI, microsatellite instability; PD‐L1 AMP, PD‐L1 amplification; TMB, tumor mutation burden; TMB‐H, high TMB

In addition to exploring the distributions of the four biomarkers in Chinese patients with a wide variety of tumor types, we also analyzed the correlations among these biomarkers. As shown in Figure 5A, the four biomarkers overlap with each other. Among 337 Chinese patients with cancer showing TMB‐H, 11.28% were also positive for dMMR, 7.4% for MSI‐H, and 2.7% for PD‐L1 AMP. In addition, nine cases in the TMB‐H cohort exhibited MSI‐H and dMMR simultaneously, while one patient was positive for all four biomarkers. Up to 76.26% of TMB‐H tumors did not coincide with any of the other biomarkers (Figure 5B). In conclusion, TMB‐H is a relatively independent biomarker with a small overlap with other biomarkers.

We also calculated the TMB values of patients with amplified and normal PD‐L1. The median values of TMB in the two groups were 94 and 95 NSM, respectively, with no significant difference between the two groups (Figure 5C). Moreover, 34.6% of PD‐L1 AMP patients were classified as TMB‐H. These results suggest that the correlation between TMB‐H and PD‐L1 AMP is small. Thus, it is necessary to simultaneously detect both to identify more suitable cancer patients for immune therapy.

**Figure 6:**
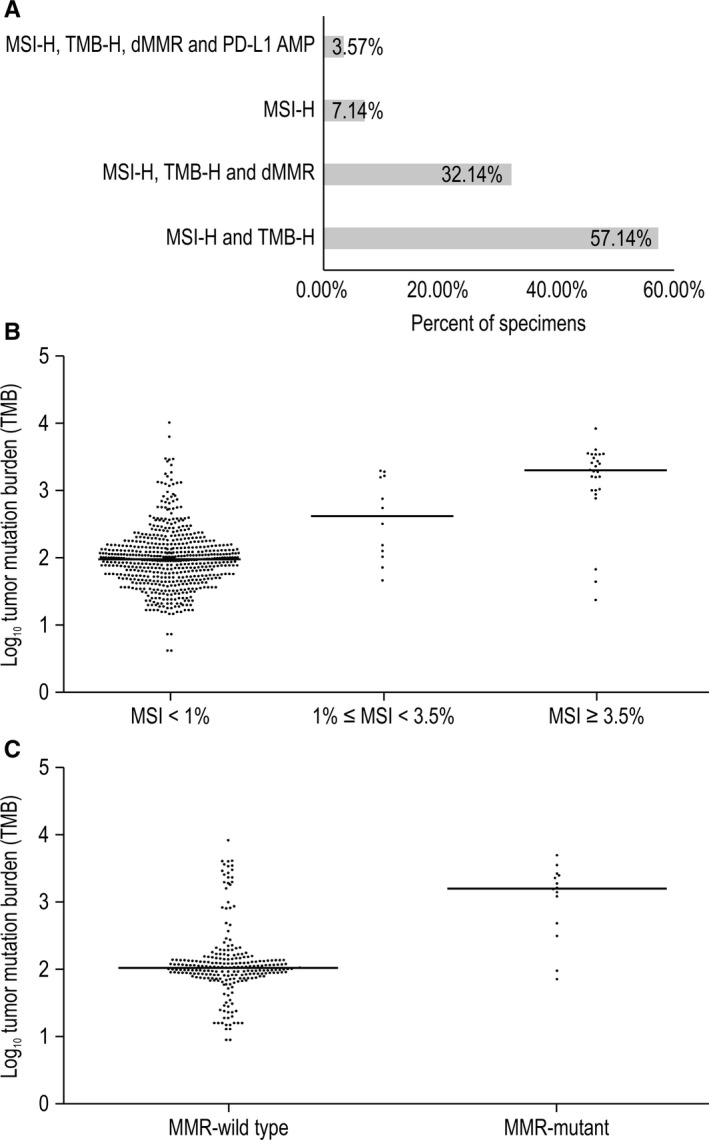
Correlations between MSI‐H and TMB‐H, dMMR, and PD‐L1 AMP. A, Proportions of MSI‐H cases with other biomarkers. B, TMB in the MSI‐H, MSI‐L, and MSS groups. Significant *P*‐values were observed using Mann‐Whitney *U* test between MSI‐H and MSI‐L (*P* = 0.0062), MSI‐H and MSS (*P* < 0.001), and MSI‐L and MSS (*P* = 0.0058). C, TMB in CRC with (n = 15) or without (n = 237) MMR mutations. *P* < 0.0001 by Mann‐Whitney *U* test. CRC, colorectal cancer; dMMR, DNA mismatch repair deficiency; MSI‐H, high MSI; MSI‐L, low MSI; MSS, microsatellite stable; TMB, tumor mutation burden; TMB‐H, high TMB

Furthermore, we investigated the correlations between MSI‐H and the other three biomarkers. MSI arises from mutations or epigenetic alterations in the MMR proteins (MLH1, MSH2, MSH3, MSH6, PMS1, or PMS2).^30^ As anticipated, we found a correlation between MSI‐H and dMMR: 32.14% of Chinese patients with cancer showing MSI‐H exhibited dMMR, and those cancer patients were TMB‐H (Figure 6A). While MSI‐H and dMMR were correlated, the association was not particularly strong, suggesting that MSI‐H may also be caused by additional factors.

High MSI tumors exhibit high levels of neoantigen, causing strong local and systemic immune responses.^31^ Therefore, it is necessary to investigate the relationship between MSI and TMB. The median TMB values of the MSI‐H, MSI‐L, and MSS groups were 1945, 408, and 90 NSM per tumor, respectively, reflecting significant differences among groups (Figure 6B). About 89% of Chinese patients with cancer exhibiting MSI‐H status were classified in the TMB‐H group.

We also found a remarkable correlation between dMMR and TMB‐H in CRC. A comparison of the TMB values of dMMR patients (n = 15) and MMR‐wild type patients (n = 237) showed that increased TMB was significantly associated with dMMR (*P* < 0.0001). dMMR tumors on average had 15‐fold more NSM (1567 vs 106 NSM) than patients with MMR‐wild type (Figure 6C).

In each of the MSI‐H and dMMR groups, there were only two cases with PD‐L1 AMP, representing rates of 7% and 4%, respectively. PD‐L1 AMP cases also exhibited low frequencies of dMMR and MSI‐H (<10% each), which is consistent with a previous report showing that among 365 solid tumors only 10.9% of the MSI‐H cases exhibited PD‐L1 AMP.^32^

## 4 DISCUSSION

To our knowledge, this is the first study characterizing TMB, MSI, dMMR, and PD‐L1 AMP in Chinese patients with more than 18 tumor types, as determined using WES. The incidence rates in TMB‐H, MSI‐H, dMMR, and PD‐L1 AMP were 35%, 4%, 0.53%, and 3.79% respectively.

Chinese patients with cancer exhibited a characteristic pattern of TMB. The two cancer types with the highest TMB were lung cancer and CRC, similar to the results of prior studies.^8^, ^26^ In contrast, hepatocellular carcinoma, breast cancer, and esophageal cancer cases exhibited TMB values 1‐3 times higher than those in TCGA cohort.^26^ The CHECKMATE‐040 and APHINITY trials showed that adjuvant treatment with nivolumab and pertuzumab effectively improved the overall response rate of hepatocellular carcinoma previously treated with sorafenib and the invasive disease‐free survival of HER2^+^ breast cancer.^33^, ^34^ For unresectable or metastatic PD‐L1‐positive triple‐negative breast cancer, the use of a combination of atezolizumab and protein‐bound paclitaxel was approved by the FDA as a first‐line treatment.^35^ This indicates that more Chinese hepatocellular carcinoma, breast cancer, and esophageal cancer patients could benefit from the PD‐1/PD‐L1 blockade and that TMB can be used as a potential immunotherapeutic biomarker.

We collected more LUAD patients than LUSC patients, and we found that the TMB values of the two types differed among Chinese patients. In contrast, the prevalence of TMB‐H among LUAD patients is similar to that among LUSC patients in TCGA.^26^ Moreover, in clinical trials, the treatment of both advanced squamous and nonsquamous NSCLC with nivolumab resulted in similar improvements over treatment with docetaxel, with overall survival rates of 42% and 51% and response rates of 20% and 19%, respectively.^2^ Similarly, in a trial with nivolumab as the first‐line treatment for advanced NSCLC, squamous and nonsquamous groups had similar median progression‐free and overall survival.^36^ Based on our findings, Chinese patients with LUSC may demonstrate a higher potential for clinical benefits of the PD‐1/PD‐L1 blockade. In addition, we found that patients with *EGFR* mutations had significantly lower median TMB compared with those with wild‐type *EGFR*, consistent with another study in which the TMB value of *EGFR*‐mutant nonsquamous NSCLC was half that of wild‐type.^37^

Moreover, the cut‐off value of TMB to predict treatment response has been determined in several studies. Another study involving melanoma and NSCLC clinical data found the cut‐off to be 192 NSM, with 74% sensitivity and 59.3% specificity.^8^ In this study, more than 20% of patients with LUSC, other subtypes of lung carcinoma, hepatocellular carcinoma, glioma, esophageal carcinoma, and CRC had NSM counts above 192, indicating that TMB‐H may be a good indicator for the use of PD‐1/PD‐L1 blockade. However, the optimal TMB cut‐offs require further exploration in clinical trials with Chinese patients with cancer to guide the use of immunotherapy.

In the current study, the frequency of tumors classified as MSI‐H was 8.3% for CRC, while lung and breast cancer exhibited MSI‐H frequencies of 0.4% and 0.66%, respectively. By contrast, a comprehensive review of studies that investigated MSI before 2014 showed that CRC exhibited MSI‐H frequencies of 13%, while those of lung cancer and breast cancer were 2% and 1%, respectively.^28^ Besides the lower rate of MSI‐H for CRC in our cohort, the frequency of MSI‐H in gastric cancer was very low (2.3%), and the rates of MSI‐H in gastric cancer patients from other countries were 18%‐22%.^28^, ^38^ This suggests that MSI‐H may not be useful for screening gastric patients before PD‐1/PD‐L1 blockade and that the efficacy of using other biomarkers or a combination of MSI‐H with other biomarkers should be investigated further.

Compared with TMB and MSI, research involving the frequency of PD‐L1 AMP in many cancer types is lacking. In this study, we fully surveyed PD‐L1 AMP in Chinese patients with cancer. We found relatively high rates in LUSC (14.3%), HER2^+^ breast cancer (8.8%), and breast cancer with unknown HER2 status (5.8%), and low rates in LUAD (1.75%) and CRC (1.59%). A study of more than 100 patients with many cancer types also surveyed the PD‐L1 AMP distribution. Sarcoma, LUSC, colorectal adenocarcinoma, and invasive breast carcinoma exhibited PD‐L1 AMP rates of over 15%, while LUAD exhibited rates of 10%‐15%.^29^ Thus, the rates of PD‐L1 AMP among Chinese patients with LUAD, breast cancer, and CRC were lower than those observed in this previous study.

While TMB, dMMR, MSI, and PD‐L1 AMP are different genetic alterations that occur in many cancers, they may be inherently related. In an analysis of 11348 cancer patients, 27% of patients with TMB‐H exhibited MSI‐H, and 70% of MSI‐H cases had high TMB.^39^ An analysis of 100 000 cancer genomes from TCGA, found that 83% of the MSI‐H samples had high TMB.^20^ By contrast, in this study, we found that Chinese cancer patients showing TMB‐H exhibited relatively low rates of MSI‐H and dMMR. On the other hand, about 89% of the Chinese cancer patients with an MSI‐H status belonged to the TMB‐H group. These results indicate that MSI‐H can cause TMB‐H, but TMB‐H is not primarily caused by MSI‐H.

We found that 32.14% of Chinese patients with cancer showing MSI‐H exhibited dMMR. The overlap between the MSI‐H and dMMR cases is higher than what was previously reported (13.37%).^38^ We also found that, on average, dMMR CRC carried 15‐fold more NSM (1567 vs 106 NSM) than CRC with MMR‐wild type, in accordance with a previous report (1782 vs 73 NSM, respectively).^13^

These findings can be interpreted based on data from a previous study regarding the hypermutation of human cancers. This study found that patients with TMB of >100 Mut/Mb may be classified as MSS, but many contain replicative polymerase mutations resulting in replication repair deficiency. Moreover, TMB values of 10–100 Mut/Mb were mostly associated with MSI‐H and had high levels of dMMR.^40^ This study, therefore, explains to a certain extent why TMB‐H, MSI‐H, and dMMR do not completely overlap and indicates that there may be several additional underlying causes of TMB‐H, for example, replicative polymerase mutations.

Moreover, a recent study has suggested that the underlying causes of these four biomarkers may differ. High TMB and MSI are caused by defects in the DNA damage repair system,^41^ which is composed of many proteins in addition to MMR components, including the homologous recombination repair element RecA/Rad51^42^ and the non‐homologous end joining repair element Ku70/Ku80.^43^ Research has shown that in the absence of MSI, mutations in DNA polymerase (POLE) can lead to TMB‐H.^44^ PD‐L1 AMP can be caused by the breakage‐fusion‐bridge cycle, extra replication, and recombination, among other mechanisms, many of which are distinct from the underlying causes attributed to the other biomarkers. This explains the minimal overlap between PD‐L1 AMP and the other biomarkers.

This study has several limitations. First, we only collected information on cancer type, while demographic and clinical characteristics such as age, gender, and tumor stage were not recorded. Therefore, we cannot conduct a more detailed subgroup or stratification analysis to obtain more clinical information, for example, investigating whether TMB is related to age and treatment type. Second, the cut‐off values for classification into the MSI‐H and TMB‐H categories were relative and were not based on the results of clinical trials. Finally, this study lacked assessment of PD‐L1 expression by immunohistochemistry. The assessment of cancer‐specific thresholds of these biomarkers by comprehensive analysis of clinical outcomes and patient characteristics would be more valuable for clinical application.

## 5 CONCLUSION

In conclusion, our study characterized the distributions of TMB, MSI, dMMR, and PD‐L1 AMP in Chinese patients with cancer and investigated the relevance of these biomarkers. Although these biomarkers could be used to identify cancer patients who may respond to immunotherapy, they cannot perfectly predict the efficacy of immunotherapy. More extensive studies investigating new biomarkers or a combination of biomarkers are therefore needed.

## ETHICS APPROVAL AND CONSENT TO PARTICIPATE

All procedures followed the Molecular Pathology Clinical Practice Guidelines and Reports^16^ and were performed in accordance with the 1964 Declaration of Helsinki. Informed consent was obtained from each of the participants.

## CONFLICT OF INTERESTS

The authors declare that they have no competing interests.

## AUTHOR CONTRIBUTIONS

SJ, YZ, and QX conceptualized the study, analyzed the data, and critically reviewed the manuscript. SJ acquired funding for the study. CD, XC, and XX collected data, designed the experiments, and administered the project. GW and JW collected and interpreted the data. BW and WS wrote the manuscript. All authors read and approved the final manuscript and agree to be accountable for the accuracy and integrity of the work.

## Data Availability

The datasets used and/or analyzed in the study are available from the corresponding author on reasonable request. The datasets generated and/or analyzed in the study are available in the BioRxiv repository: https://www.biorxiv.org/search/Comprehensive%252Banalysis%252Bof%252Bpotential%252Bimmunotherapy%252Bgenomic%252Bbiomarkers%252Bin%252B1%252C000%252BChinese%252Bpatients%252Bwith%252Bcancer.
